# Unveiling Forkhead-mediated regulation of yeast cell cycle and metabolic networks

**DOI:** 10.1016/j.csbj.2022.03.033

**Published:** 2022-04-07

**Authors:** Matteo Barberis, Thierry D.G.A. Mondeel

**Affiliations:** a Systems Biology, School of Biosciences and Medicine, Faculty of Health and Medical Sciences, University of Surrey, Guildford, Surrey GU2 7XH, UK; b Centre for Mathematical and Computational Biology, CMCB, University of Surrey, Guildford, Surrey GU2 7XH, UK; c Synthetic Systems Biology and Nuclear Organization, Swammerdam Institute for Life Sciences, University of Amsterdam, Amsterdam 1098 XH, The Netherlands

**Keywords:** Systems biology, Networks, Transcriptional regulation, Forkhead transcription factors, Cell cycle, Metabolism

## Abstract

Transcription factors are regulators of the cell’s genomic landscape. By switching single genes or entire molecular pathways on or off, transcription factors modulate the precise timing of their activation. The Forkhead (Fkh) transcription factors are evolutionarily conserved to regulate organismal physiology and cell division. In addition to molecular biology and biochemical efforts, genome-wide studies have been conducted to characterize the genomic landscape potentially regulated by Forkheads in eukaryotes. Here, we discuss and interpret findings reported in six genome-wide Chromatin ImmunoPrecipitation (ChIP) studies, with a particular focus on ChIP-chip and ChIP-exo. We highlight their power and challenges to address Forkhead-mediated regulation of the cellular landscape in budding yeast. Expression changes of the targets identified in the binding assays are investigated by taking expression data for Forkhead deletion and overexpression into account. Forkheads are revealed as regulators of the metabolic network through which cell cycle dynamics may be temporally coordinated further, in addition to their well-known role as regulators of the gene cluster responsible for cell division.

## Highlights

• Findings from genome-wide ChIP studies on budding yeast Forkheads are interpreted.
• Power, challenges and limitation of ChIP studies are presented by target gene analysis.
• Forkheads regulate metabolic targets through which cell division may be coordinated.

## 1 Introduction

The interplay of intracellular signals that regulate gene expression occurs at the Transcription Start Site (TSS) of genes, where signals are integrated through a complex of proteins including the RNA polymerase. Transcription factors are proteins that bind, relatively close to the TSS, to specific DNA sequence elements (‘motifs’) that typically span 5–12 base pairs. They then interact with histones and other transcription factors on the DNA, thus impacting the assembly of a TSS protein complex. In addition, enhancer elements may influence transcription distantly from a TSS.

Mapping the binding sites of transcription factors across the genome in living cells has been made possible through Chromatin Immunoprecipitation (ChIP), the first genome-wide methodology with microarray detection of bound DNA (ChIP-chip) [1]. This technique relies on: (i) the use of formaldehyde to chemically link proteins and DNA together, (ii) the sonication of the DNA to fragment it, (iii) the purification of a selected protein by an antibody, and (iv) the detection of DNA fragments bound to the purified protein. The first attempt to identify, through this methodology, the sequence elements that are bound by transcription factors was shown for the *Saccharomyces* species [2]. With the emergence of next generation sequencing, ChIP followed by high-throughput DNA sequencing (ChIP-seq) was developed, which achieved a higher resolution as compared to ChIP-chip because of not being limited by the amount of probes on the chip [3]. Of note, ChIP-seq has been employed to map a large number of human transcription factors with a consistent set of experimental and data processing protocols [4]. A more recent development in the ChIP methodology is ChIP-exo, which further ameliorates ChIP-seq by using a lambda exonuclease digestion, to degrade one strand of the isolated DNA, followed by high-throughput sequencing [5]. ChIP-exo allows for the identification of binding sites at promoters with near-single-nucleotide accuracy [6].

ChIP data provide a list of targets (i.e. genomic locations where binding occurs) of transcription factors that may be subsequently tested, thus ‘predicting’ their possible novel cellular functions. In the analyses of ChIP data, false-positives binding events have to be identified, in order to retrieve a comprehensive, but reliable picture of a transcription factor’s functions. Transcription factors may bridge multiple spatial, temporal and functional scales across cellular layers of regulation, such as cell cycle, metabolism, signalling, etc. Thus, they may be hubs, i.e. nodes in a network characterized by a high connectivity, at the interface between cellular layers in multi-scale models that aim to understand how biological functions emerge from networks of interactions [7].

Forkhead (Fkh) transcription factors are highly conserved across eukaryotes. In humans, they play a role in a number of cellular pathways that, when dysregulated, may lead to development of pathologies such as cancer [8], [9], neurodegeneration [10], [11], and aging [12], [13]. In budding yeast, Forkheads control organismal physiology by regulating the cluster of genes responsible for cell division [14] and by modulating the precise transcription timing of replication origins. Dynamics of DNA replication are realized as these molecules bind to [15], [16], are rate-limiting activators of [17], and are responsible for the clustering of [18] DNA replication origins. Of note, a differential effect on individual origins was observed upon deletion of either Fkh1 or Fkh2, with Forkheads-activated origins being most frequently bound by Fkh1 only and generally not bound by Fkh2 only [16].

Here, we focus on the complexity of the ChIP-based interaction landscape that has recently emerged for the yeast Forkheads. Specifically, we point out both the validity and uncertainty of target genes retrieved for the transcription factors Fkh1 and Fkh2.

## 2 Forkheads integrate cell division with multi-scale physiology

Microarray-based RNA profiling [19] and ChIP-chip studies using DNA microarrays [20] have retrieved a wide spectrum of Fkh1 and Fkh2 target genes [16], [21], [22]. The latter experiments were conducted growing cells at a similar optical density (OD): 0.8 [16], likewise 0.8 [21] (the experimental work being originally performed in [2]), and 1.0 [22]. Among these targets, several metabolic enzymes were identified, suggesting a possible function of the yeast Fkhs in cellular processes beside regulation of cell division.

Recently, we have shown that the yeast Fkhs targets promoters of novel target genes, among which cell cycle genes as well as genes involved in metabolism and signal transduction [23]. By using the ChIP-exo methodology and developing a novel data analysis method called *maxPeak* – which is not sensitive to a relatively low number of strong peaks obtained by the ChIP-exo as compared to other existing “peak detection methods” such as GEM [24] and MACE [25], also employed in the study – we have provided the most comprehensive overview of the current knowledge of Fkh target genes in budding yeast. By integrating the ChIP-exo pipeline with the information about functional annotation, timing and RNA transcript levels of target genes, Fkh targets that scored above threshold in at least two out of three peak detection methods among *maxPeak*, GEM, and MACE were selected. Our analyses provided the high-confidence genes whose expression may be modulated by Fkh1 and Fkh2 [23].

Well-known Fkh targets involved in cell cycle control were retrieved: (i) the *CLB2* gene whose transcription levels peak in the early mitotic phase (G2/M transition) of the cell cycle to control cell division through the activity of Clb2/Cdk1 kinase complexes [26], and (ii) the *SWI5* gene [27], [28] which activates the transcription of genes expressed in the late mitotic phase (M/G1 transition) of the cell cycle. The Fkh-mediated tipping of the balance between a biochemical activator (Clb2/Cdk1) – which phosphorylates and stimulates the degradation of Sic1 [29] – and a biochemical inhibitor (Sic1) – which inhibits the Clb2/Cdk1 activity [30] – governs the precise timing of cell division. Furthermore, other cell cycle regulators have been identified as Fkh targets, for which a dedicated experimental validation is not yet available. Among these there are: the cyclin gene *CLB1* that promotes cell division [31], [32], together with its cognate *CLB2*; the cyclin gene *CLN1* that promotes budding events [33], [34], together with its cognate *CLN2*; and the transcription factor *ACE2* that promotes *SIC1* transcription [35], together with *SWI5*.

Intriguingly, transcription factors and metabolic enzymes that play a role in central carbon metabolism and are crucial for cell growth and division were also identified as Fkh targets [23]. Single mutants of some of the metabolic enzymes result in a reduced growth rate [36], pointing to a potential function of Fkh1 and Fkh2 in metabolic events.

After our study, two new binding studies have been recently published that report on the spectrum of targets of transcription factors in budding yeast among which Fkh1 and Fkh2 [37], [38]. In the first study, the ChIP-exo/seq methodology was employed to explore the architecture of chromatin-associated proteins with a high-resolution [37]. In the second study, gene regulatory variations that alter transcription factors binding were investigated through the ChEC methodology [38], a chromatin endogenous cleavage that uses fusion of a protein of interest to a micrococcal nuclease (MNase) to target calcium-dependent cleavage to specific genomic loci *in vivo* [39]. We have examined the datasets available from these studies together with the high-throughput datasets that we have previously analyzed [16], [21], [22], [23], thus combining six binding studies. In addition, we have incorporated the results of single-gene deletion [40] and overexpression time course [41] experiments, which may be used to functionally validate potential targets from the binding studies.

To further explore the potential relevance of Forkheads in the regulation of yeast cellular networks, among which cell cycle and metabolic processes, we first thoroughly analysed the six binding studies, i.e. three ChIP-chip using DNA microarrays [16], [21], [22], two ChIP-exo [23], [37], and one ChEC [38], for retrieving the consensus of Fkh1 and Fkh2 target genes. We then merged the consensus data from these binding studies to the deletion / overexpression studies with the information we collected through GEMMER, a web-based data-integration and visualization tool that we have recently developed to integrate and visualize the large experimental data available for budding yeast [42]. The Saccharomyces Genome Database (SGD) (https://www.yeastgenome.org/) was queried – following engagement with SGD curators who have conducted a dedicated update of the YeastMine database [43] including previously missing ChIP data from [16], [23] – and data was extracted. SGD currently only contains the list of target genes from [22] that activate specifically under heat-shock and not otherwise. Of note, we expanded on the data in SGD by also including targets shown in the 25UTmax experiment from [22] in the GEMMER database.

**Fig. 1:**
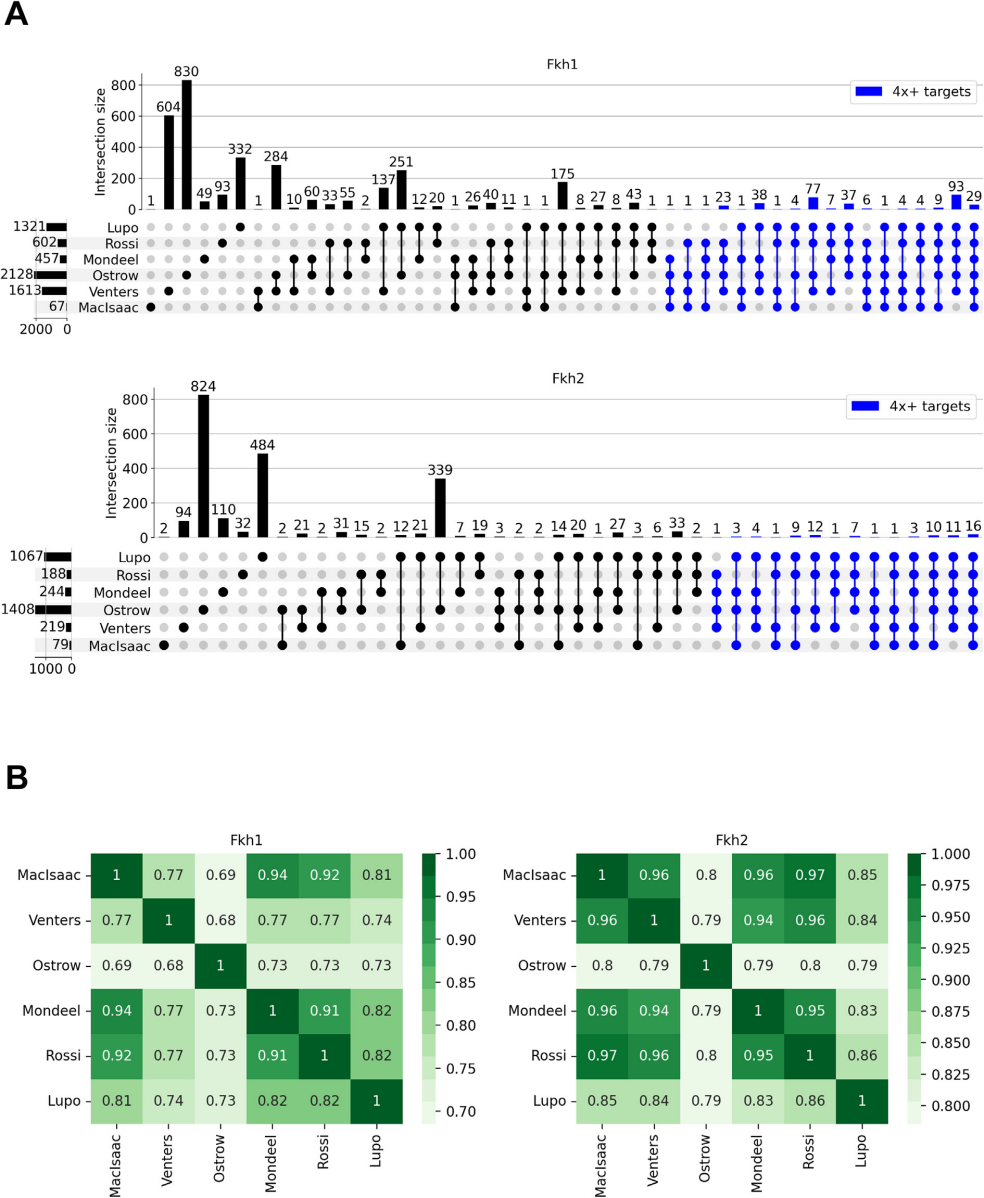
A) UpSet plot for Fkh1 and Fkh2 target genes. All intersections between the six binding studies that contain at least one gene are shown. The barplot on the bottom-left indicates the number of target genes in each study. The barplot on top indicates the number of genes in a particular intersection. Black dots represent the studies considered for a particular intersection. Intersections of four or more studies are highlighted in blue. B) Hamming similarity matrices for the six binding studies for Fkh1 and for Fkh2.

Supplementary Excel Table 1 summarizes the results of the merging procedure, collecting the complete set of information that forms the basis for our analysis. The six binding studies can be summarized as Boolean vectors indicating whether a gene is considered a target, and similarity metrics can be calculated to compare those. Because of the infeasibility to plot intersections of six datasets as a Venn diagram with complete coverage, we show the overlap of targets in the form of an UpSet plot [44] in Fig. 1A. A Hamming similarity matrix counts the fraction of genes for which two datasets agree (either both true or both false). By using this metric, the datasets of MacIsaac [21], Mondeel [23], and Rossi [37] reveal the highest similarity for Fkh1, whereas MacIsaac [21], Mondeel [23], Venters [22], and Rossi [37] reveal the highest similarity for Fkh2 (Fig. 1B). This result mainly stems from the large number of targets reported in the studies of Ostrow [16], Venters [22] (for Fkh1), and Lupo [38] (using the thresholds we have set to the data; see Supporting Information).

As we highlighted in our previous study [23], binding studies (and even different peak detection methods) for Fkh1 and Fkh2 are highly variable, as it can be observed in Fig. 1A. Thus, it may be unrealistic to expect functional targets to be revealed in all six binding datasets. We therefore inspected in detail only those targets retrieved by at least four out of six binding studies. The selection returns 337 targets of Fkh1 and 80 targets of Fkh2. Gene annotation performed through GEMMER reveals that metabolic processes are largely represented as potential targets of Fkhs, followed by cell cycle/cell division and signal transduction (see Supplementary Excel Table 1).

The deletion and overexpression studies [40], [41] (using the thresholds we set to the data; see Supporting Information) provide information about whether Fkh1 and Fkh2 have an impact on genes across the genome. However, it is not *a priori* clear that all functional targets should respond to both deletion and overexpression of each transcription factor. In fact, alternative transcriptional regulators may exist that can be involved in the regulation of a specific gene (e.g. Fkh1 and Fkh2 have overlapping functions, and take over the function of one another in the absence of either gene [45]), or cofactors may be required as rate-limiting molecules in response to the transcription factor overexpression (e.g. the chromatin binding of the coactivator Ndd1 is required for the periodic activity observed for Fkh1 and Fkh2 transcripts [46]). We therefore restricted the following step of our analysis to the targets retrieved by more than four binding studies (4x+) that respond to either deletion or overexpression or both. Selecting those targets that respond to either deletion or overexpression already disregards a sizable percentage of the 4x+ targets. Here we refer to the target genes satisfying criteria in both validation studies as ‘fully validated’, and to the set of genes satisfying one or both of the criteria as ‘(partially) validated’.

**Fig. 2:**
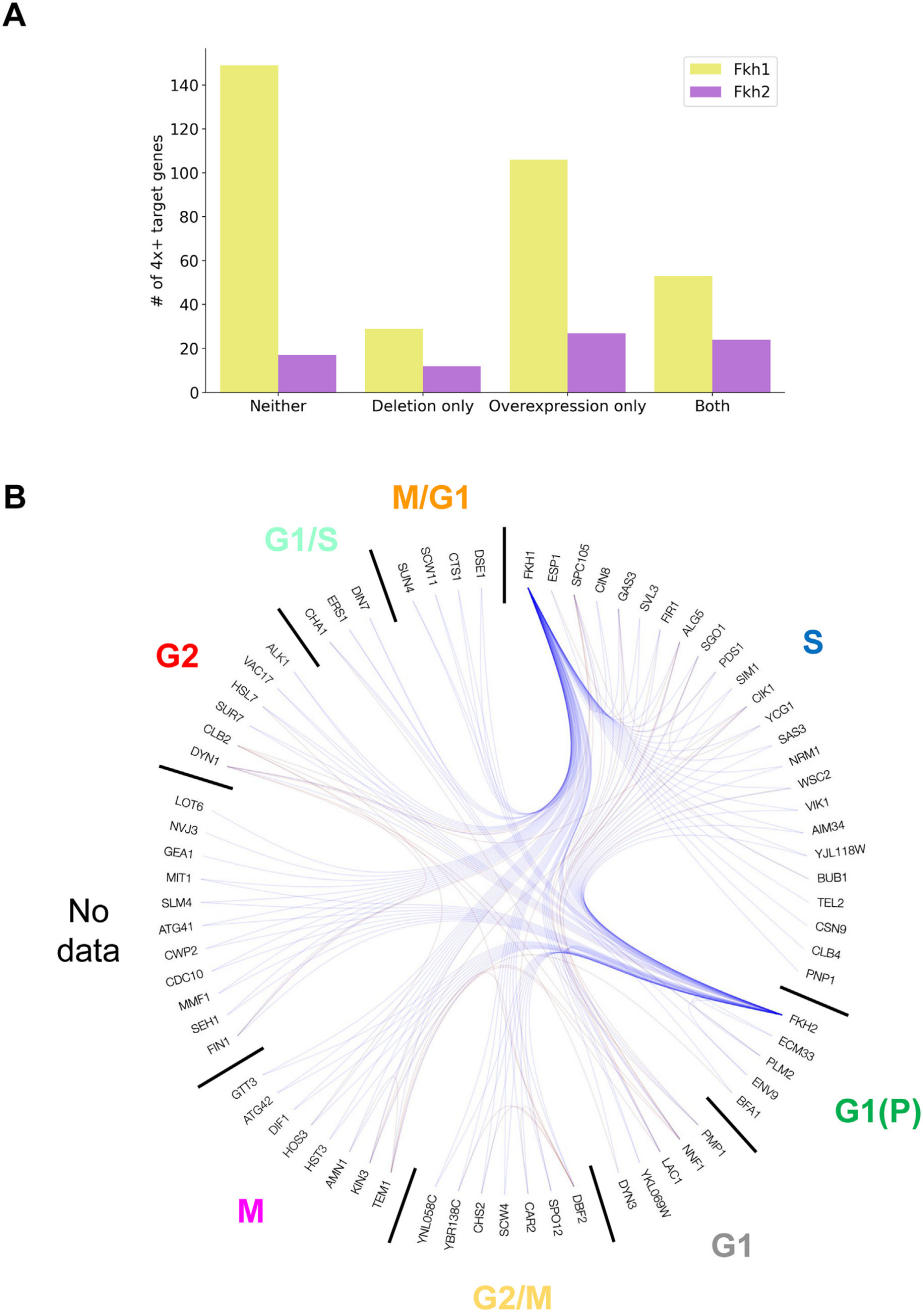
Regulatory (gene targets) and physical (protein–protein) interactomes associated to the Forkhead transcription factors (Fkhs) Fkh1 and Fkh2 in budding yeast. A) Barplot of Fkhs target genes shared by at least four (4x+) ChIP studies across four categories with respect to their response to deletion and overexpression experiments (see Supporting Information). B) Hierarchical edge bundling plot of the 71 Fkhs target genes shared by 4x+ ChIP studies which also respond to both deletion and overexpression experiments. Both regulatory (blue) and physical (red) interactions are shown. Targets are clustered by phase of peak expression (if available) for cell cycle-regulated genes, according to [47]. From the top-right quadrant, clockwise starting from Fkh1, the phases are: S, G1(P), G1, G2/M, M, No data, G2, G1/S, M/G1.

Fig. 2A summarizes the result of the deletion and overexpression experiments for the 4x+ target genes of Fkh1 and Fkh2. The set of (partially) validated 4x+ genes comprises 188 Fkh1 targets and 63 Fkh2 targets (see Supplementary Excel Table 1). Among these genes, 53 Fkh1 targets and 24 Fkh2 targets are fully validated as they respond to both overexpression and deletion of Fkhs. Fig. 2B displays a hierarchical edge bundling plot of the fully validated 4x+ target genes of Fkh1 and Fkh2, clustered according to the phases of peak expression for cell cycle-regulated (CCR) genes as described in a genome-wide dataset of gene expression [47] (see Supplementary Excel Table 2 for the details about the regulatory interactions).

Interestingly, among this fully validated set for Fkh2, all 24 target genes were downregulated at the end of the overexpression time course, two genes were first upregulated and then downregulated, and only one gene (*ATG42*) was upregulated. Similarly, 47 out of 53 fully validated Fkh1 targets were downregulated at the end of the overexpression time course, four of which were upregulated. Conversely, and in agreement with the overexpression experiments, the deletion experiments revealed that most of these target genes were upregulated (40 out of 53 for Fkh1 and 20 out of 24 for Fkh2). An exception is *CLB2*, the major Fkh target gene. *CLB2* is – as expected – downregulated upon Fkh2 deletion; however, notably, it first rises before dropping below wild type levels in the overexpression time course. This result suggests that the genes transcriptionally regulated after *CLB2* activation (the so-called *CLB2*-cluster [14]) are promptly transcribed upon Fkh2 activation to trigger cell division; these genes are then switched off upon *CLB2* deactivation, for the cell to re-enter into a new cell cycle round.

**Fig. 3:**
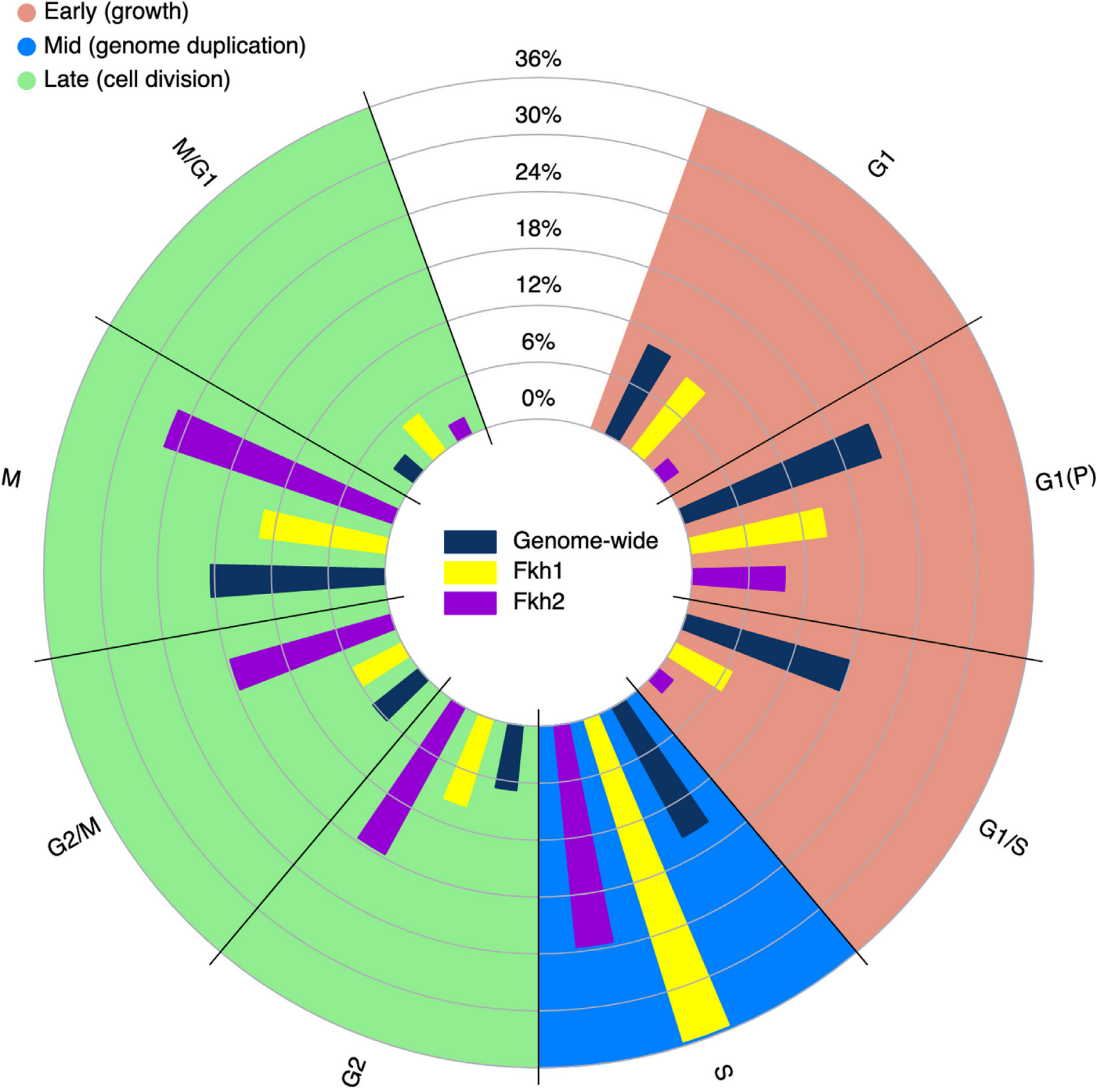
Distribution of peak expression for all cell cycle-regulated (CCR) genes and for the (partially) validated CCR Fkh1 and Fkh2 targets in the various cell cycle phases: Early, growth (red); Mid, genome duplication (blue); and Late, cell division (green).

Among the (partially) validated 4x+ target genes, 104 out of 188 Fkh1 targets and 51 out of 63 Fkh2 targets are CCR genes [47]. The fraction of CCR genes is much higher than across the whole genome, where it is less than 15%. Both Fkh1 and Fkh2 have cell cycle regulated targets that peak in all phases of the cell cycle; however, here we show that their relative proportions across the phases differ from the genome-wide distribution as well as from one another. Fig. 3 highlights the under- and overrepresentation of (partially) validated CCR targets per cell cycle phase for Fkh1 and for Fkh2 as compared to the genome-wide phase distribution. The peak of expression of Fkh1 and Fkh2 targets is relatively absent (less than 15% of CCR genes) in the early (G1, G1(P), G1/S) phases – during cell growth –, whereas their targets peak (more than 20% of CCR genes) in the middle (S) phase – during genome duplication. Strikingly, a distinct activation of Fkh1 and Fkh2 functions is observed, with: Fkh1 targets being strongly overrepresented in the middle (S) phase, dropping in the late (M) phase, and becoming overrepresented again (5% of CCR genes) in the late (M/G1) phase of a new cell cycle; and Fkh2 targets being overrepresented (between 18 and 25% of CCR genes) in both middle (S) and late (G2, G2/M, M) phases.

This distribution suggests a specificity of function for Fkh1 and Fkh2 throughout cell cycle progression, which might be maintained and/or enhanced through the physical interactome of Fkh1 and of Fkh2, and of their target genes. Yet, Fkh1 and Fkh2 share 34 (partially) validated 4x+ target genes in common (*AMN1*, *ASE1*, *ATG42*, *BUD4*, *CAR2*, *CIN8*, *CLB2*, *CTS1*, *CWP2*, *DIF1*, *ENV9*, *FRK1*, *GIC1*, *GLN1*, *HOS3*, *HSL7*, *IDI1*, *KIP2*, *RNR1*, *RPL37B*, *SED1*, *SGO1*, *SIM1*, *SLM4*, *SNA2*, *SRL1*, *TEM1*, *VAC17*, *WSC2*, *WSC4*, *WTM1*, *YBR138C*, *YCG1*, *YHP1*) – more than half of the Fkh2 targets – that are mostly confined in the replicative phases of the cell cycle, specifically in S, G2, G2/M and M, with the majority in S and M phases (Supplementary Excel Table 1). This result confirms the reported overlapping role between the two Fkhs [45].

In the fully validated 4x+ target genes (Supplementary Excel Table 2), the majority of genes (around 40%) associated to both Fkh1 and Fkh2 has a function in ‘Cell cycle’ and/or ‘Cell division’ (GO term 1) (Supplementary Excel Table 3), as expected for pivotal regulators of cell division. Of note, around 20% of genes associated to both Fkhs has a function in ‘Metabolism’ (GO term 1) (Supplementary Excel Table 3). Accordingly, among the (partially) validated 4x+ target genes, around 30% (56 out of 188) of Fkh1 targets and around 20% (14 out of 63) of Fkh2 targets are metabolic enzymes. Thus, Fkh1 targets a higher number of metabolic genes than Fkh2, both relatively and absolutely. Supplementary Excel Table 4 summarizes the KEGG pathways associated to the (partially) validated 4x+ target genes. Noteworthy, seven among the Fkh1 targets play a role in central carbon metabolism: *ACS2*, *ADH1*, *ADH4*, *PDC1* (glycolysis); *RPE1* (pentose phosphate pathway); and *CIT1*, *CIT2* (citrate/TCA cycle). Of note, these targets were all retrieved in the analyses we carried out earlier [23], [48].

Among the targets, the metabolic enzymes *ATG42*, *HOS3* and *SIM1* are fully validated CCR targets for both Fkh1 and Fkh2. Strikingly, *ATG42* and *HOS3* are found as targets in all the six binding studies and are fully validated with respect to the deletion and overexpression experiments.

**Fig. 4:**
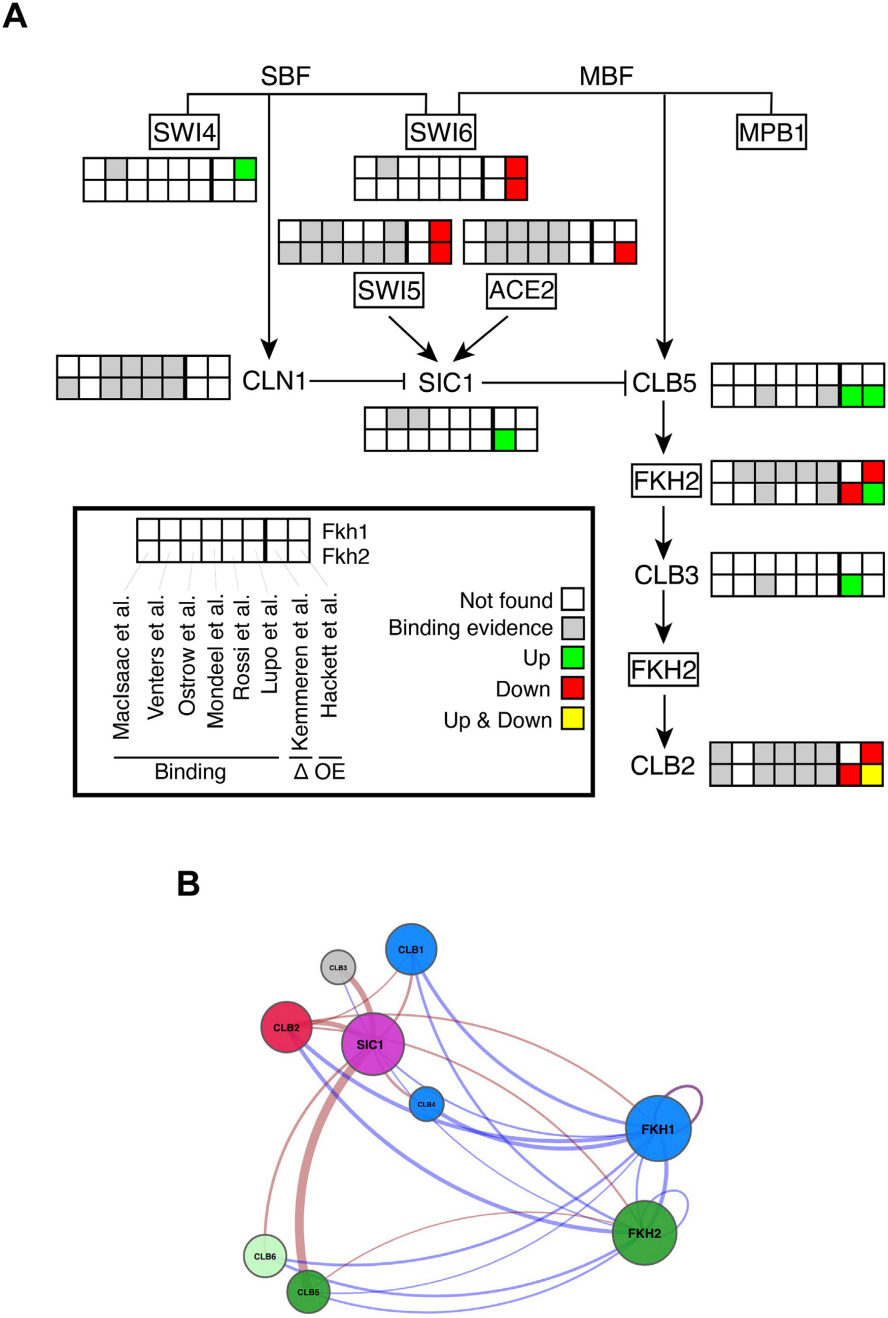
Fkh1 and Fkh2 target genes in the molecular cascade regulating dynamics of cell cycle progression. A) Overview of cell cycle regulators that are Fkh targets. The transcription factors *SWI4*, *SWI6*, *MPB1*, *SWI5*, *ACE2* and *FKH2* are shown within rectangles. B) Regulatory (blue) and physical (red) interactions are plotted through GEMMER [42] within the minimal network of the mitotic cyclin/Cdk activity, where Fkh1, Fkh2, mitotic cyclins (Clb1-Clb6) and their stoichiometric inhibitor Sic1 are shown. Interactions with one or more ChIP studies are shown. Target genes are colored by phase of peak expression and clustered using the wild type #2 of the CYCLoPs database (Cyclops WT2) [66]. Because no CYCLoPs data is available for Clb5 and Clb6, they form their own cluster.

Altogether, our analyses reveal that Forkhead transcription factors are hubs that connect intracellular pathways, in particular metabolism and cell cycle, operating at different but specific times. The integration of signals may modulate Fkhs-mediated gene expression, on top of their regulatory interactome that emerges from the six binding studies for the genes coding for the mitotic cyclins (the G1/S cyclins *CLB5* and *CLB6* and the G2/M cyclins *CLB1*–*CLB4*) and their M/G1 stoichiometric inhibitor *SIC1* (Fig. 4 and Supplementary Excel Table 5). Strikingly, this minimal network of the Forkhead-centred mitotic cyclin/Cdk1 activity can coordinate temporal dynamics of cell division in the budding yeast cell cycle, as we have shown both experimentally and computationally [49], [50].

## 3 Predictive power and challenges of ChIP-based Forkhead studies

The ChIP studies conducted on Fkhs, including the most recent efforts using the ChIP-exo methodology [23], [37], match a number of independent experimental analyses. Five out of six studies show an enrichment of Fkh2 at the *CLB2* promoter; accordingly, *CLB2* mRNA levels are reduced upon Fkh2 deletion [40], [50]. Furthermore, one of the ChIP-chip studies reports an enrichment of Fkh2 at a small region of promoters of both *CLB5* and *CLB3* cyclin genes [16]. While Clb5 promotes timely initiation of DNA replication, early steps in the formation of mitotic spindles and cell cycle arrest upon DNA damage [51], [52], [53], [54], Clb3 promotes spindle assembly and elongation [32], [55]. Of note, an opposite outcome is observed in an independent validation study for *CLB5* and *CLB3* levels upon deletion of Fkh2, with *fkh*2Δ cells exhibiting reduced *CLB3* mRNA levels but not *CLB5* mRNA levels [50]. This evidence suggests a different affinity of Fkh2 for the *CLB* promoter regions, yet to be experimentally investigated, which may result in a selective *CLB* activation. This regulatory mode, together with the activation of Fkh2 through phosphorylation mediated by the progressive accumulation of successive Clb/Cdk1 complexes [50], [56], may be in place to timely transcribe *CLB* genes. This mechanism can guarantee self-sustained, autonomous oscillations of Clb/Cdk1 activities [57], [58], thereby the unidirectionality of cell cycle progression.

Conversely, ChIP studies highlight some incongruences with respect to the molecular mechanisms that have been validated through independent molecular biology and biochemical investigations. Though a Systems Biology, integrative strategy combining predictive mathematical modeling and dedicated experimental testing, we have recently discovered a role of Fkh2 as temporal coordinator of mitotic Clb waves, identifying Fkh2 as a controller molecule of the sequential activation of *CLB* expression [50]. Specifically, Fkh2 binds to *CLB3* promoter, positively regulating Clb3 expression, which in turn contributes to the Fkh2-dependent transcription of *CLB2* in a linear cascade (Clb5 → Clb3 → Clb2) [50]. This result contradicts the early study of Kemmeren which shows *CLB3* being upregulated upon Fkh2 deletion [40]. However, Fkh2 binding to *CLB3* promoter is only shown by one of the ChIP-chip studies [16]. In fact, in our early study [23], *CLB3* does not score above the stringent threshold imposed in any of the three peak detection methods (*maxPeak*, GEM, and MACE) employed to analyze ChIP-exo data, thus resulting in its exclusion from being retrieved as Fkh2 target. This case indicates that genes exhibiting a low DNA binding signal in ChIP studies should not be disregarded as potential targets but require further investigation through independent experimental testing.

On the one hand, the high stringency that we have used to analyze ChIP data calls for an experimental validation of the high-scoring target genes. On the other hand, it does not confirm previously identified Fkh targets through ChIP-chip, such as *SIC1* for Fkh1 [22], [16] and *CLB5* for Fkh2 [16]. In line with the latter finding, we have shown that Fkh2 deletion does not affect productively *CLB5* expression levels [49]. Similarly, the binding of Fkh1 to *CLB4* promoter in a genome-wide location analysis [59] has been only partially confirmed by our independent analyses. Specifically, although we did not detect Fkh1 binding to *CLB4* promoter, Fkh1 deletion resulted in an increased *CLB4* transcription and in a change of RNA pol II occupancy [50], suggesting an indirect regulation of Clb4, yet to be elucidated. Of note, two ChIP-chip studies showed an enrichment of Fkh1 at multiple, overlapping binding sites at both the *CLB4* promoter region and *CLB4* ORF [2], [16]. This evidence, together with the fact that we do not observe a binding of Fkh1 to *CLB4* promoter, leads to suspect a lower affinity that this multiple, overlapping binding sites at *CLB4* promoter may have for Fkh1.

This observation finds a parallel with a ChIP-based genome-wide study of human Fox transcription factors, which have revealed that an extensive overlap in chromatin binding can dictate their recruitment to chromatin [60]. The study has pointed to a scenario where the human Fkhs may control gene expression through dynamic binding at the same DNA-binding site, rather than through a mutually exclusive binding of specific Fox molecules [60]. This evidence further supports the suspect that a lower affinity of Fkhs at binding sites on the DNA may be a conserved mechanism across eukaryotes to regulate plasticity of gene regulation dynamics.

## 4 Perspectives

Altogether, the evidence presented here for the Fkhs in budding yeast suggests that complementary studies, from genome-wide, ChIP-based studies to independent molecular biology- and biochemistry-based experiments are needed to exclude false positives and point to the effective targets of transcription factors. Specifically, to interpret ChIP data, identification of stable method(s) for data analysis accuracy in the identification of functional targets shall be taken into account.

In humans, the FoxM1 and FoxP transcription factors are homologs of the yeast Fkh1 and Fkh2 [61], and FoxM1 regulates the expression of the mitotic Cyclin B [62] through a similar mechanism by which Fkh2 regulates Clb2 expression [63]. Furthermore, human and yeast Fkhs share the core sequence of a DNA-binding motif that is recognized by other members of the human Forkhead family [64], and also matches part of the motif that we and others have identified for Fkh1 and Fkh2 [21], [23]. The similarity in both the binding motif and the actual targets between the human and yeast Fkhs suggests that target genes retrieved through genome-wide, ChIP-chip and ChIP-exo studies may be conserved in various cellular pathways across the two organisms.

ChIP-based methodologies have the power to generate hypotheses, which predictions may be directly tested experimentally through molecular biology and biochemical investigations. Moreover, this data can be integrated into computational approaches [65] that explore the role of transcriptional regulation for the cell’s temporal dynamics. Importantly, the conservation across eukaryotes of Forkhead (Fkh) transcription factors as molecules linking different layers of cellular networks suggests that their role is pivotal in the timely regulation of vital processes to guarantee cell’s functions. Therefore, punctual investigation of Fkh targets may shed new light on organismal functions that require coordination of precise timing of cell cycle events with multi-scale physiology.

## Funding

This work was supported by the Systems Biology Grant of the 10.13039/501100003513University of Surrey to M.B.

## CRediT authorship contribution statement

**Matteo Barberis:** Conceptualization, Methodology, Validation, Formal analysis, Investigation, Resources, Data curation, Writing – original draft, Writing – review & editing, Visualization, Supervision, Project administration, Funding acquisition. **Thierry D.G.A. Mondeel:** Methodology, Formal analysis, Resources, Data curation, Writing – review & editing, Visualization.

## Declaration of Competing Interest

The authors declare that they have no known competing financial interests or personal relationships that could have appeared to influence the work reported in this paper.

## Data Availability

The data shown in this article are available as Supplementary Information. Source code for the analysis is available through a Github repository (https://github.com/barberislab/Forkhead-mediated_regulation).
