# Supplementary material for: Unveiling Forkhead-mediated regulation of yeast cell cycle and metabolic networks
Source: Comput Struct Biotechnol J. 2022 Apr 7;20:1743–51. doi: 10.1016/j.csbj.2022.03.033 (PMC9024378; doi:10.1016/j.csbj.2022.03.033)
Supplement: Supplementary data 1 [file mmc1.pdf]

**SUPPORTING INFORMATION**

# Unveiling Forkhead-mediated regulation of yeast cell cycle and metabolic networks

Matteo Barberis and Thierry D. G. A. Mondeel

## METHODS

The analysis was performed by merging the datasets of target genes for six ChIP studies (MacIsaac [1], Venters [2], Ostrow [3], Mondeel [4], Rossi [5], and Lupo [6]) and by using GEMMER (http://gemmer.barberislab.com) [7]. The resulting dataset was filtered considering the deletion [8] and overexpression time course [9] experiments for Fkh1 and Fkh2.

The targets identified in three out of the six available binding studies (MacIsaac [1], Ostrow [3], Mondeel [4]) are contained in the Saccharomyces Cerevisiae Database (SGD) (https://yeastmine.yeastgenome.org/yeastmine/begin.do). GEMMER, which in part relies on the SGD database, contains these results as well. Furthermore, GEMMER contains the target genes found in the remaining three binding studies (Venters [2], Rossi [5], and Lupo [6]). The latter two were manually added in preparation for this manuscript.

Targets from Venters [2] were based on the results of the 25C UTmax experiment from that publication, using the p-value thresholds as indicated in their Supplementary Table S4a. Data from Rossi [5] was taken from the Supplementary Data 2 of that publication, specifically the "All_sectors_x_all_targets" sheet from where the columns for Fkh1 and Fkh2 were extracted. Data from Lupo [6] was obtained through personal correspondence with the authors; the data from the non-hybrid *Saccharomyces cerevisiae* experiments for Fkh1 and Fkh2 were used for the analysis. In [6], a threshold was not used to decide on whether a gene was a target or not; instead, the top 100 genes ranked by signal were discussed. Starting from the genome-wide dataset of [6], the list of targets was defined as all genes that had a z-score > 0. Due to the non-negativity of the signal distribution, the dataset is right-skewed and the mean is larger than the median. As a result, the z-score > 0 criteria selects less than 20% of genes. All source files can be found in the Github repository for this publication. Deletion data [8] was obtained from https://deleteome.holstegelab.nl through the downloadable file “deleteome_all_mutants_controls.txt” using the M values (which are log2(deletion mutant / wild type) ratios) and the p-values for our analysis. Overexpression

time course data [9] was obtained through the Supplementary datasetEV1 from that publication.

Deletion [8] and overexpression time course [9] experiments were used to validate potential Fkh1 and Fkh2 targets suggested by the six binding studies. Both studies report on log2 fold-changes compared to wild type. Since functional targets are expected to show an effect in these experiments, a “(partially) validated target gene” was defined as a gene showing an effect in either the overexpression and/or deletion experiments for each Forkhead transcription factor. In the deletion experiments, a gene was considered a target gene when it shows a fold-change of 1.7 and is statistically significant at a p-value threshold of 0.05 [8]. Since [8] regarded hundreds of transcription factors, this stringent criteria was useful to control false-positives. However, in our analysis, a reduced stringency is sought. Specifically, the significance threshold used in the deletion study was kept, and the 1.7 fold-change threshold was used to label target genes as either ’strongly up’ or ‘strongly down’. In addition, genes with a fold-change > 1.1 were labeled as either ‘up’ or ‘down’, and genes with a fold-change < 1.1 were labeled as ‘weakly up’ or ‘weakly down’. This last threshold was chosen, in part, because the well-known Fkh2 target gene *CLB2* scores just above this threshold. We therefore considered any target gene scoring ‘up/down/strongly up/strongly down’ or passing the significance threshold as ‘validated’. In other words, the validation threshold for the deletion experiment was for a gene to be statistically significant and/or have a fold-change of at least 1.1. The overexpression experiments involve a time course of Fkh1 and Fkh2 levels measured at 0, 5, 8, 15, 30, 45, 60 and 90 minutes after induction [9]. The pre-processing of the time courses used in the overexpression study resulted in most of the protein-coded genes – as we report in our analysis – being “invariant”, i.e. not responding to overexpression (e.g. only around 20% – 1417 out of 6807 – genes showed any response for Fkh1). All responsive genes showed a fold-change of at least 1.1 in at least one time-point. When looking only at timepoints with a fold-change > 1.1, the target gene time courses were classified as ‘up’ or ‘down’, ‘strongly up’ or ‘strongly down’ or a combination of either. The

‘strongly up/down’ classification was applied when at least one time point shows a fold-change > 2. For the overexpression experiments, any gene that was not invariant was considered as validated.

**REFERENCES**


1. MacIsaac KD, Wang T, Gordon DB, Gifford DK, Stormo GD, Fraenkel E. An improved map of conserved regulatory sites for Saccharomyces cerevisiae. BMC Bioinformatics 2006;7;113.
2. Venters BJ, Wachi S, Mavrich TN, Andersen BE, Jena P, Sinnamon AJ, et al. A comprehensive genomic binding map of gene and chromatin regulatory proteins in Saccharomyces. Mol Cell 2011;41;480–492.
3. Ostrow AZ, Nellimoottil T, Knott SR, Fox CA, Tavaré S, Aparicio OM. Fkh1 and Fkh2 bind multiple chromosomal elements in the S. cerevisiae genome with distinct specificities and cell cycle dynamics. PLoS One 2014;9;e87647.
4. Mondeel TDGA, Holland P, Nielsen J, Barberis M. ChIP-exo analysis highlights Fkh1 and Fkh2 transcription factors as hubs that integrate multi-scale networks in budding yeast. Nucleic Acids Res 2019;47;7825–7841.
5. Rossi MJ, Kuntala PK, Lai WKM, Yamada N, Badjatia N, Mittal C, et al. A high-resolution protein architecture of the budding yeast genome. Nature 2021;592;309–314.
6. Lupo O, Krieger G, Jonas F, Barkai N. Lupo O. Accumulation of cis- and trans-regulatory variations is associated with phenotypic divergence of a complex trait between yeast species. G3 (Bethesda) 2021;11;jkab016.
7. Mondeel TDGA, Crémazy F, Barberis M. GEMMER: GEnome-wide tool for Multi-scale Modeling data Extraction and Representation for Saccharomyces cerevisiae. Bioinformatics 2018;34;2147–2149.

8. Kemmeren P, Sameith K, van de Pasch LA, Benschop JJ, Lenstra TL, Margaritis T, et al. Large-scale genetic perturbations reveal regulatory networks and an abundance of gene-specific repressors. Cell 2014;157;740–752.
9. Hackett SR, Baltz EA, Coram M, Wranik BJ, Kim G, Baker A, et al. Learning causal networks using inducible transcription factors and transcriptome-wide time series. Mol Syst Biol 2020;16;e9174.
